# Skin microcirculatory reactivity assessed using a thermal challenge is decreased in patients with circulatory shock and associated with outcome

**DOI:** 10.1186/s13613-018-0393-7

**Published:** 2018-05-03

**Authors:** Diego Orbegozo, Wasineenart Mongkolpun, Gianni Stringari, Nikolaos Markou, Jacques Creteur, Jean-Louis Vincent, Daniel De Backer

**Affiliations:** Department of Intensive Care, Erasme University Hospital, Université Libre de Bruxelles, Route de Lennik 808, 1070 Brussels, Belgium

**Keywords:** Skin blood flow, Skin laser Doppler, Capillary blood flow, Perfusion, Nitric oxide, Peripheral circulation, Laser Doppler flowmetry

## Abstract

**Background:**

Shock states are characterized by impaired tissue perfusion and microcirculatory alterations, which are directly related to outcome. Skin perfusion can be noninvasively evaluated using skin laser Doppler (SLD), which, when coupled with a local thermal challenge, may provide a measure of microcirculatory reactivity. We hypothesized that this microvascular reactivity would be impaired in patients with circulatory shock and would be a marker of severity.

**Methods:**

We first evaluated skin blood flow (SBF) using SLD on the forearm and on the palm in 18 healthy volunteers to select the site with maximal response. Measurements were taken at 37 °C (baseline) and repeated at 43 °C. The 43 °C/37 °C SBF ratio was calculated as a measure of microvascular reactivity. We then evaluated the SBF in 29 patients with circulatory shock admitted to a 35-bed department of intensive care and in a confirmatory cohort of 35 patients with circulatory shock.

**Results:**

In the volunteers, baseline SBF was higher in the hand than in the forearm, but the SBF ratio was lower (11.2 [9.4–13.4] vs. 2.0 [1.7–2.6], *p* < 0.01) so we used the forearm for our patients. Baseline forearm SBF was similar in patients with shock and healthy volunteers, but the SBF ratio was markedly lower in the patients (2.6 [2.0–3.6] vs. 11.2 [9.4–13.4], *p* < 0.01). Shock survivors had a higher SBF ratio than non-survivors (3.2 [2.2–6.2] vs. 2.3 [1.7–2.8], *p* < 0.01). These results were confirmed in the second cohort of 35 patients. In multivariable analysis, the APACHE II score and the SBF ratio were independently associated with mortality.

**Conclusions:**

Microcirculatory reactivity is decreased in patients with circulatory shock and has prognostic value. This simple, noninvasive test could help in monitoring the peripheral microcirculation in acutely ill patients.

**Electronic supplementary material:**

The online version of this article (10.1186/s13613-018-0393-7) contains supplementary material, which is available to authorized users.

## Background

Circulatory shock is a life-threatening condition affecting about one-third of patients admitted to the intensive care unit (ICU) [1, 2]. Regardless of the underlying pathophysiological mechanisms, the hallmark of shock states is altered tissue perfusion, which if not rapidly corrected leads to organ dysfunction and death [3, 4]. Recent data have highlighted the prognostic importance of microcirculatory abnormalities in patients with shock using noninvasive bedside techniques, such as sublingual video-microscopy [5–8]. The hallmark of these alterations is decreased capillary density, and microvascular blood flow with increased heterogeneity of perfusion [9, 10]. Interestingly, these microcirculatory abnormalities are not explained by routinely measured global macro-hemodynamic variables, making it attractive to assess them directly [11, 12].

The microcirculation can also be studied by evaluating the response generated by a hypoxic stress event, such as during a transient vascular occlusion test (VOT) [7, 8]. Near-infrared spectroscopy (NIRS) or laser Doppler techniques can be used to indirectly or directly evaluate a transient increase in flow after a VOT [13–15]. Studies using these techniques have shown that endothelial reactivity is impaired in sepsis and is associated with organ dysfunction and outcome [16, 17]. However, VOTs are not easily standardized (duration of occlusion and/or tissue oxygen saturation [StO_2_] reached) [16, 18]. In addition, VOTs may alter local metabolism [19]. Alternative methods to evaluate microvascular recruitment are, therefore, of interest.

Local heating of the skin may represent an alternative means of evaluating microvascular reactivity. Skin laser Doppler (SLD) (also known as laser Doppler flowmetry) can be used to assess skin blood flow (SBF) during a thermal challenge [14]. This technique uses an optical fiber to direct light from a low-power laser source to the skin and to collect the back-scattered light. The shift in light wavelength is proportional to the red blood cell velocity in the studied skin area, providing a noninvasive measurement of SBF expressed as arbitrary perfusion units (PUs) [20]. New SLD flow probes can heat the explored tissue in a controlled way, making it possible to perform a dynamic test of capillary reactivity by increasing local temperature [14]. However, there are no published data evaluating this test in patients with circulatory shock.

We hypothesized that reactivity of the skin microcirculation, evaluated as the skin blood flow ratio, during a thermal challenge would be impaired in patients with circulatory shock. We also assessed whether these alterations could be explained by other hemodynamic parameters and were correlated with patient outcome.

## Methods

This prospective, observational study was conducted in our 35-bed Department of Intensive Care. Institutional Ethical Committee approval was obtained, and informed consent was obtained from each participant or the next of kin.

### Protocol

To assess the most appropriate probe position for an SLD thermal challenge, we first studied 18 healthy volunteers. They were comfortably seated in a quiet, temperature-controlled room for at least 15 min before each experiment. Heart rate, respiratory rate and hemoglobin saturation were evaluated noninvasively by pulse oximetry using a Siemens SC 9000 monitor (Siemens, Erlangen, Germany). Noninvasive measurements of mean arterial pressure (MAP) were taken in the opposite arm to that used for the SLD blood flow measurements.

We then studied, in 2013, a cohort of 29 critically ill adult patients admitted with a diagnosis of circulatory shock, defined as the need for norepinephrine infusion to maintain a MAP of at least 65 mmHg, associated with an altered mental status, acute oliguria defined as a urine output < 0.5 ml/Kg/h or an arterial lactate level > 2 mmol/L [4]. Screening of ICU admissions, collection of data and SLD measurements were performed by doctors not involved in patient management. All SLD measurements were taken as soon as possible after completion of initial resuscitation, i.e., when an adequate arterial pressure for that patient had been reached (determined by the treating physician), and norepinephrine doses had been stable for 1 h. A second SLD measurement was taken, when possible, 48 h after the first measurement.

Having analyzed the results from our first patient cohort, we repeated the study in a second, confirmatory cohort of critically ill patients with circulatory shock (using the same definition) admitted in 2015. Patients were evaluated by an investigator who had not been involved in the initial study and at just one time point during the first day of hospitalization after initial resuscitation.

We collected demographic and clinical data on admission and classified patients as having sepsis or not, based on standard criteria [21]. At the time of each SLD measurement, we collected all available hemodynamic and respiratory data from ongoing patient monitors and recorded the central body temperature. We also collected biochemical and laboratory data from clinical records, including the most recent blood gas analysis. The APACHE II score [22] was calculated using the worst data during the first 24 h in the ICU. The sequential organ failure assessment (SOFA) score [23] was calculated from the data present at the time of the SLD measurements. Patients were grouped according to ICU outcome (dead or alive) for further analysis.

### Skin laser Doppler measurements

All SLD measurements were taken using the PeriFlux System 5000 monitor (Perimed, Jarfalla, Sweden), and data were continuously collected (PeriSoft software 2.5.5; Perimed) for further analysis. For the thermal challenge, a small angled thermostatic SLD probe 457 (Perimed) with 0.25 mm fiber separation was used. This probe also allows skin temperature measurement at site of application. The SLD machine emits a beam of laser light with a wavelength of 780 nm that allows skin evaluation at a depth of 0.5–1 mm. The initial skin temperature was measured with the thermostatic probe prior to each measurement. In the healthy volunteers, the probe was placed on the skin of the volar face of the proximal forearm and on the palm of the same arm. In the patients, the SLD probe was placed on the forearm without the arterial line. The probe was kept in position using the double-sided tape provided with the SLD monitor. All participants were asked to abstain from any activity during the study period to prevent any possible artifacts in the recorded signals [13].

To limit differences between subjects in the basal temperature, SBF was recorded at a local skin temperature of 37 °C allowing for at least three minutes of stabilization. Thereafter, a thermal challenge was performed by increasing the probe temperature abruptly (0.1 °C/s) from 37 to 43 °C. After 9 min at 43 °C, the SBF was recorded (Additional file 1: Figure S1). We chose to increase the temperature to 43 °C, because our preliminary experience indicated that using lower thresholds (39 °C or 41 °C) reduced the amplitude of the response, making it more difficult to detect potential differences. We calculated the SBF ratio (SBF obtained at 43 °C/SBF obtained at 37 °C) as a simple measure of microvascular reactivity in the explored area.

### Statistical analysis

Statistical analyses were performed using SPSS 22.0 (IBM, New York, NY) software. Variables were assessed for normality of distribution using skewness and kurtosis tests and Q–Q plots. Continuous variables are presented as means ± standard deviations or median values with percentiles (25–75%) depending on the presence or absence of normality. Categorical data are presented as numbers of events and percentages. Repeated measurements were compared using a paired Student’s *t* test or Wilcoxon signed rank test, as appropriate. Comparisons between different cohorts were made using an unpaired *t* test or Mann–Whitney *U* test as appropriate. Proportions were compared with a Chi-square test or Fisher’s exact test as appropriate. We plotted the sensitivity and specificity using a receiving operating characteristics (ROC) graph, and the area under the curve (AUC) was calculated for the different variables as a measure of their ability to predict mortality. To assess possible explanatory variables correlated with the SBF ratio, we plotted individual data on graphs and calculated the Pearson or Spearman correlation coefficient (*r*) as appropriate. Univariate and multivariate analyses (binary logistic regressions) were performed to identify the ability of different variables to predict ICU mortality, calculating the odds ratios and their respective 95% confidence intervals.

In a post hoc analysis considering that the APACHE II score was directly correlated and the SBF ratio inversely correlated with mortality, we calculated the ratio between the two factors (APACHE II score/SBF ratio) in an attempt to improve their prognostic value.

A two-sided *p* value less than 0.05 was considered as significant for all analyses.

## Results

### Healthy volunteers

**Fig. 1:**
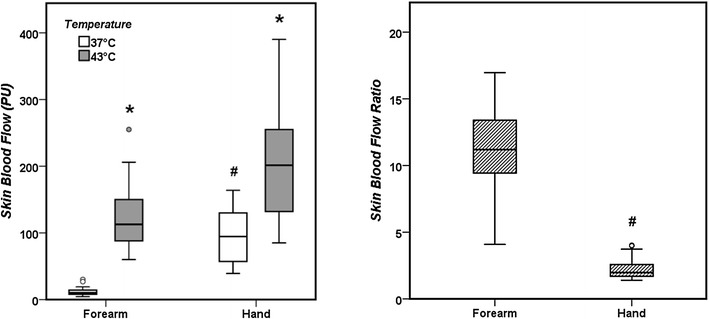
Comparison of skin blood flow measurements at different temperatures on the forearm and the hand in healthy volunteers. In the right panel, the ratio of skin blood flow obtained between 43 and 37 °C is presented. *p < 0.05 compared with measurements at 37 °C; #*p* < 0.05 compared with forearm measurements

The main characteristics of the volunteers are listed in Additional file 1: Table S1. Comparisons of measurements taken in the hand and the forearm are shown in Fig. 1 and Additional file 1: Table S2. Baseline values of SBF at 37 °C were lower in the forearm than in the hand, but the SBF ratio was higher in the forearm.

### Patients: initial cohort

**Fig. 2:**
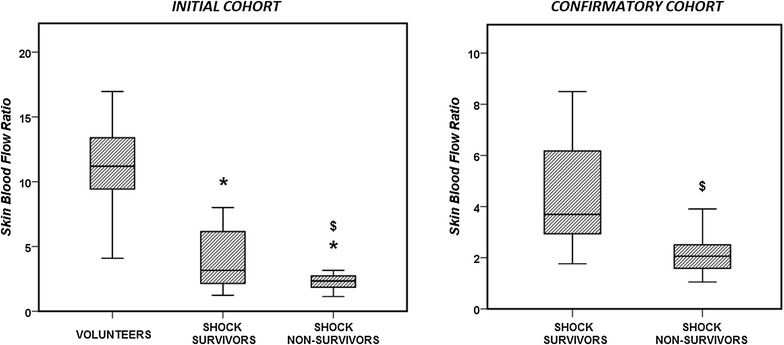
Comparison of the capillary reactivity, evaluated as the skin blood flow ratio, in the healthy volunteers and the patients with circulatory shock (initial and confirmatory cohorts). *p < 0.05 compared with healthy volunteers; $*p* < 0.05 compared with survivors

The main demographic and baseline characteristics of the 29 patients are listed in Table 1: 16 (55%) had septic and 13 (45%) cardiogenic shock. Eleven patients died in the ICU (44%). Times from ICU admission until the first measurement were similar in survivors and non-survivors: 6 (4–17) versus 7 (5–14) h (*p* = 0.59). The patients had a similar baseline SBF to the healthy volunteers, but a lower SBF at 43 °C, which resulted in a lower SBF ratio (Table 2). SBF values at baseline and at 43 °C were similar in survivors and non-survivors, but survivors had significantly higher SBF ratios (Table 2). A comparison of the SBF ratios in volunteers, survivors and non-survivors is shown in Fig. 2.

**Table 1:** Main baseline characteristics of patients with circulatory shock

Variable	Initial cohort				Confirmatory cohort			
	Total (*n* = 29)	Survivors (*n* = 18)	Non-survivors (*n* = 11)	*P*	Total (*n* = 35)	Survivors (*n* = 17)	Non-survivors (*n* = 18)	*P*
Age (years)	67 ± 11	64 ± 12	71 ± 8	0.11	64 ± 16	61 ± 16	67 ± 16	0.30
Body mass index (kg/m^2^)	26 ± 6	25 ± 5	27 ± 8	0.66	28 ± 5	27 ± 5	28 ± 5	0.29
Septic shock [*n* (%)]	16 (55)	9 (50)	7 (64)	0.82	28 (80)	13 (77)	15 (83)	0.61
Cardiogenic shock [*n* (%)]	13 (45)	9 (50)	4 (36)	0.82	7 (20)	4 (23)	3 (17)	0.61
Chronic kidney disease [*n* (%)]	8 (28)	7 (39)	1 (9)	0.11	8 (23)	4 (24)	4 (22)	1.00
Chronic arterial hypertension [*n* (%)]	20 (69)	11 (61)	9 (82)	0.41	11 (31)	5 (29)	6 (33)	0.80
Diabetes mellitus [*n* (%)]	12 (41)	7 (39)	5 (46)	0.73	11 (31)	5 (29)	6 (33)	0.80
Coronary artery disease [*n* (%)]	9 (31)	5 (28)	4 (36)	0.69	8 (23)	3 (18)	5 (28)	0.69
Mean arterial pressure (mmHg)	75 ± 13	73 ± 9	77 ± 17	0.52	73 ± 9	72 ± 7	75 ± 10	0.28
Heart rate (bpm)	97 ± 25	99 ± 25	95 ± 27	0.67	99 ± 22	101 ± 22	98 ± 22	0.71
Central venous pressure (mmHg)	11 (8–12)	11 (7–12)	11 (9–13)	0.28	9 (7–12)	8 (6–11)	10 (8–12)	0.23
Cardiac output (L/min)	4.0 (3.4–5.7) *n* = 18	3.8 (3.2–4.7) *n* = 11	4.1 (3.5–6.8) *n* = 7	0.44	4.8 (3.5–6.3) *n* = 32	4.8 (3.4–6.7) *n* = 16	4.6 (3.6–5.6) *n* = 16	0.68
Central temperature (°C)	36.8 (36.0–37.5)	37.1 (36.2–37.8)	36.5 (35.9–37.3)	0.19	36.9 (36.6–37.5)	36.8 (36.6–37.2)	37.0 (36.6–37.6)	0.71
Basal forearm temperature (°C)	32.0 (31.1–32.7)	32.1 (31.1–32.7)	31.4 (30.7–32.7)	0.58	30.6 (29.1–31.6)	30.5 (28.9–31.6)	30.7 (30.0–31.8)	0.55
Central to basal forearm temperature (°C)	5.1 (4.2–6.1)	5.0 (4.5–6.4)	5.0 (4.2–5.6)	0.44	6.5 (5.6–7.6)	7.4 (5.2–7.9)	6.4 (5.8–7.1)	0.54
pH	7.35 ± 0.07	7.36 ± 0.06	7.33 ± 0.09	0.29	7.35 ± 0.12	7.33 ± 0.13	7.36 ± 0.11	0.42
PCO_2_ (mmHg)	38 ± 8	39 ± 9	35 ± 8	0.22	37 ± 11	40 ± 14	35 ± 8	0.22
Lactate (mmol/L)	2.4 (1.6–4.8)	1.9 (1.5–2.9)	3.2 (1.6–6.3)	0.35	1.7 (1.2–2.6)	1.6 (1.1–2.7)	1.8 (1.3–2.4)	0.78
Mechanical ventilation [*n* (%)]	25 (86)	15 (83)	10 (91)	1.00	25 (71)	10 (59)	15 (84)	0.11
PaO_2_/FiO_2_ ratio	192 (132–293)	196 (128–310)	192 (145–250)	0.93	166 (124–244)	189 (124–247)	146 (120–211)	0.24
Creatinine (mg/dL)	1.4 (1.0–1.7)	1.4 (0.9–1.6)	1.6 (1.0–2.2)	0.44	1.3 (0.8–2.0)	1.3 (0.8–1.9)	1.2 (0.8–2.1)	0.82
Total bilirubin (mg/dL)	0.9 (0.5–2.1)	0.9 (0.5–1.8)	1.6 (0.6–2.8)	0.30	0.8 (0.5–2.7)	0.8 (0.5–2.7)	0.9 (0.5–2.6)	0.58
Platelets (× 10^3^/µL)	163 (92–243)	160 (92–243)	163 (65–307)	0.93	52 (13–79)	58 (25–108)	22 (7–67)	0.06
Leukocytes (cells × 10^3^/µL)	12.4 (8.5–17.7)	11.7 (8.2–17.7)	12.4 (8.5–17.7)	0.95	12.0 (8.5–19.9)	12.5 (10.4–21.9)	10.8 (4.7–17.0)	0.16
Number of patients receiving sedatives [*n* (%)]	17 (59)	10 (56)	7 (64)	0.67	8 (23)	4 (24)	4 (22)	1.00
Number of patients receiving opiates [*n* (%)]	22 (76)	14 (78)	8 (73)	1.00	7 (20)	4 (24)	3 (17)	0.69
APACHE II score	26 ± 8	23 ± 7	30 ± 8	0.03	26 ± 8	22 ± 8	29 ± 6	< 0.01
SOFA score	10 ± 3	10 ± 3	12 ± 3	0.04	10 ± 5	9 ± 5	11 ± 4	0.13
Number of patients receiving dobutamine [*n* (%)]	13 (45)	7 (39)	6 (54)	0.41	6 (17)	2 (24)	2 (11)	0.40
Dobutamine dose at moment of thermal challenge (mcg/Kg/min)	0 (0–10)	0 (0–5)	10 (0–15)	0.24	0 (0–0)	0 (0–0)	0 (0–0)	0.42
Norepinephrine dose at moment of thermal challenge (mcg/Kg/min)	0.32 (0.11–0.50)	0.28 (0.11–0.41)	0.43 (0.11–0.84)	0.43	0.12 (0.08–0.55)	0.12 (0.07–0.41)	0.20 (0.08–0.62)	0.36
ICU length of stay (days)	8.0 (3.5–16.0)	8.6 (4.4–16.0)	6.5 (2.1–19.6)	0.44	5.3 (3.2–9.0)	4.7 (4.0–9.0)	6.0 (3.2–8.0)	0.70

**Table 2:** Comparison of skin laser Doppler (SLD) variables between volunteers and patients and between survivors and non-survivors

SLD variable	Healthy volunteers and initial cohort			Initial cohort			Confirmatory cohort		
	Volunteers (*n* = 18)	Patients (*n* = 29)	*P*	Survivors (*n* = 18)	Non-survivors (*n* = 11)	*P*	Survivors (*n* = 17)	Non-survivors (*n* = 18)	*P*
Skin blood flow 37 °C (PU)	10.3 (8.0–14.2)	13.0 (10.1–19.5)	0.16	13.4 (10.0–21.6)	13.0 (10.1–19.5)	0.44	12.4 (8.0–17.3)	15.0 (10.2–19.3)	0.44
Skin blood flow 43 °C (PU)	112.8 (88.0–150.0)	36.4 (25.6–76.9)	< 0.01	51.8 (25.6–77.2)	33.7 (16.5–39.0)	0.76	57.4 (41.5–71.5)	33.1 (21.8–41.6)	< 0.01
Skin blood flow ratio	11.2 (9.4–13.4)	2.6 (2.0–3.6)	< 0.01	3.2 (2.2–6.2)	2.3 (1.7–2.8)	< 0.01	3.7 (2.9–6.2)	2.1 (1.6–2.5)	< 0.01

*PU* perfusion units

SLD measurements were repeated at 48 h in 20 of the patients. At that time point, the survivors had a somewhat higher SBF ratio than the non-survivors although the differences were not statistically significant [3.1 (2.4–4.3) vs. 2.3 (1.5–3.1), *p* = 0.08] (Additional file 1: Figure S2). The difference in the SBF ratio between T0 and T48 was not significant in survivors or non-survivors (Additional file 1: Figure S3).

There were no significant correlations between the SBF ratio and any hemodynamic or blood gas-derived variable, administration of vasoactive drugs (Additional file 1: Figure S4), presence of sepsis (Additional file 1: Table S3), degree/severity of organ dysfunction (SOFA score), initial forearm temperature or the difference between the central to forearm temperature (Additional file 1: Figure S5).

**Fig. 3:**
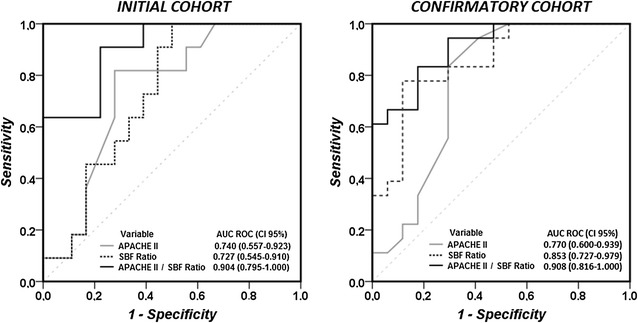
Receiver operating characteristic (ROC) curves for the predictive value of the APACHE II score, the skin blood flow (SBF) ratio and the APACHE II/SBF ratio (initial and confirmatory cohorts)

The SBF ratio and the APACHE II score had similar ROC AUCs to predict survival (0.73 [0.55–0.91] vs. 0.74 [0.56–0.92], respectively) (Fig. 3). There was no significant correlation between the SBF ratio and the APACHE II score (*r* = 0.047, *p* > 0.05), so that we calculated their combined ratio, which resulted in a larger ROC AUC to predict survival than that for either measure alone (0.90 [0.79–1.00], Fig. 3).

Considering a cutoff ratio of 3 for the SBF ratio, the specificity and sensitivity were 91 and 56%, respectively, to predict survival. Considering a cutoff value of 2 for the SBF ratio, the specificity and sensitivity were 36 and 83%, respectively.

In multivariable analysis, taking into consideration the SOFA score, the blood lactate concentration, the mean arterial pressure, the norepinephrine dose and the presence of sepsis, only the APACHE II score and the SBF ratio were independently correlated with mortality (Additional file 1: Table S4).

### Patients: confirmatory cohort

The demographic and baseline characteristics of the 35 patients in the confirmatory cohort are listed in Table 1: 28 had septic (80%) and 7 cardiogenic (20%) shock. Eighteen patients died in the ICU (51%). SBF values at baseline were similar in survivors and non-survivors, but survivors had significantly higher SBF values at 43 °C and larger SBF ratios (Table 2).

There were no significant correlations between the SBF ratio and any hemodynamic or blood gas-derived variable, dose of vasoactive drugs, presence of sepsis (Additional file 1: Table S3), degree/severity of organ dysfunction (SOFA score), initial forearm temperature or the difference between the central to forearm temperature (all *p* values > 0.05).

The ROC AUCs to predict survival were 0.85 (0.73–0.98) and 0.77 (0.60–0.94) for the SBF ratio and the APACHE II score, respectively. The ratio between the APACHE II score and the SBF ratio resulted in an ROC AUC to predict survival of 0.91 (0.82–1.00) (Fig. 3).

Considering a cutoff ratio of 3 for the SBF ratio, the specificity and sensitivity to predict survival were 83 and 71%, respectively. Considering a cutoff value of 2 for the SBF ratio, the specificity and sensitivity for prediction of survival were 50 and 88%, respectively.

## Discussion

In this study, reactivity of the skin microcirculation during a thermal challenge was compromised in patients with circulatory shock, and this impairment was related to outcome. This phenomenon could not be explained by other hemodynamic variables and was independent of regularly used scoring systems, such as the SOFA and the APACHE II score.

As far as we know, this is the first report to assess the skin microvascular reactivity (or vasodilatation) during a thermal challenge in shock patients. Several previous reports have used an ischemic challenge to assess microcirculatory reactivity [7] and shown that post-occlusive reactive hyperemia is impaired in patients with sepsis and septic shock, and this has been correlated with poor outcomes [15–17, 24]. However, this condition is not pathognomonic of sepsis and is also present in hemorrhagic [25] and cardiogenic [19, 26] shock. Our data also show that the degree of impairment in microvascular reactivity during a thermal challenge was similar in septic and cardiogenic shock. Nevertheless, studies in patients with just cardiogenic, septic or hemorrhagic shock may be of interest to further investigate whether the effects of a thermal challenge are indeed similar across different shock etiologies. Our results were not correlated with other regularly used parameters, but this is not surprising considering the considerable previous literature showing dissociation between the macro- and microcirculations [11, 12].

Importantly, an ischemic challenge can significantly alter local metabolism. Hence, some NIRS-derived variables, such as the descending slope (the rate of decrease in tissue oxygen saturation during a VOT and correlated with tissue oxygen consumption), may be affected by some degree of ischemic preconditioning in the studied tissue, limiting the reproducibility of results over a short time period [19]. The thermal challenge that we propose allows the SBF to return to baseline within minutes, enabling the test to be repeated in the same area. If there are concerns about alterations in local metabolism, the challenged area is very small (compared to the complete extremity involved during a VOT), so that the test can be repeated in an adjacent area.

The mechanisms underlying a thermal challenge are different to those of an ischemic challenge. As the skin temperature increases, the SBF increases until a maximum plateau value is reached [27–29]. More specifically, an initial short-lived phase of rapid vasodilation, which is neurally driven, is followed by a more gradual but protracted increase in SBF that is dependent on local nitric oxide (NO) production stimulated by endothelial NO synthase [27–31]. Thus, the use of this very simple and noninvasive test may provide an indirect assessment of the local NO pathway. NO plays a major role in the local control of the microcirculation and in its interaction with red blood cells [32, 33]. It also plays an important role in the pathophysiology of sepsis and septic shock [34, 35] and of cardiogenic shock [36, 37]. Interestingly, during endotoxin infusion in healthy volunteers, recruitment of the skin microcirculation through a thermal challenge that involves NO-dependent pathways was impaired, whereas post-occlusive reactive vasodilation was not [38]. Thus, the worse outcome in patients with shock who had compromised skin microvascular reactivity after the thermal challenge may reflect altered NO pathways. Nevertheless, physiological or basic science studies need to be conducted to verify the role of the NO pathway in our observations.

Two important technical aspects should be discussed further: the position of the probe on the forearm and the duration of the thermal challenge. Intuitively, the skin area used to perform SLD with a thermal challenge should have the highest potential recruitment even if the baseline SBF is relatively low. SLD studies have shown that the cutaneous microcirculation in healthy volunteers is very heterogeneous with lower blood flow values in the forearm than in the palm and lower values in the metacarpal spaces than in the fingertips [14, 39]. Similar observations have been made for other areas of the body, such as the torso, feet and face, and this heterogeneity has been confirmed by other techniques evaluating skin perfusion [40–44]. When performing a thermal challenge in different cutaneous regions, Metzler-Wilson et al. [40] showed that recruitment was higher in the forearm than in the palm. We confirmed this finding in our healthy volunteers.

Different temperature thresholds and heating times have been proposed when performing a thermal challenge [28, 29, 38, 40, 41]. However, 30 min may be needed to reach the maximal SBF during the test [28, 29], a period that is too long in critically ill patients who often need rapid changes in therapy. During pilot studies (data not shown), we observed that the best temperature to demonstrate a response was 43 °C. We also observed that we were not able to identify the initial short-lived phase of rapid vasodilation in all patients (this does not mean that the neural stimulus was absent, but just difficult to track with this technology), but when present it had already disappeared at 9 min and its analysis was difficult to standardize (time to peak versus maximum peak versus slope). For these practical reasons, we arbitrarily chose to limit the duration of the thermal challenge to 9 min. Hence, we did not evaluate the maximal recruitment of the skin microcirculation, but a surrogate value (the slope or speed of the microcirculation recruitment) within a reasonable observation period.

Other limitations should also be acknowledged. First, we included a relatively small number of patients, potentially limiting the statistical power of our analyses and possibly accounting for the lack of significant difference in microvascular reactivity between survivors and non-survivors, particularly at 48 h. Moreover, although we found similar results in our two cohorts, external validation of our data is still necessary. Second, although the forearm is a very accessible area in most critically ill patients and has the additional benefit of high potential recruitability with a thermal challenge, studies using different skin locations may also be informative. Further studies are also required to describe the evolution of these alterations over time and their changes with specific therapies (fluid bolus, transfusions, change in vasopressor dose, etc.). Although we focused on mortality as an outcome measure, future studies could also specifically assess relationships with other clinical outcomes, including changes in SOFA scores over time. Third, the presence of fever may represent a confounding factor that should be taken into account; however, the local temperature on the forearm was always less than 37 °C and there was no apparent correlation with the resulting SBF ratio. Fourth, we did not collect data from a control cohort of elderly patients (a factor that can influence the vascular response); however, our control group was designed to provide a picture of the normal physiological response and, more importantly, our main findings are related to the differences between survivors and non-survivors. Fifth, we did not compare the results of the thermal challenge to results from other concomitant measures of the microcirculation. However, our recent observations indicate that VOTs can induce ischemic preconditioning and alter local metabolism [19], which would need to be taken into account if a skin thermal challenge was performed in the same area. Finally, although it is impossible to extrapolate our skin measurements to other microvascular networks in different organs, the strong association we found with outcomes and the simplicity of this technique makes it attractive for future research.

## Conclusion

SLD with a thermal challenge is a technique that allows simple, noninvasive evaluation of skin microcirculatory reactivity. Using this technique, we have demonstrated that reactivity of skin microcirculation during a thermal challenge is compromised in patients with circulatory shock, is related to outcome and combination with the APACHE II score can improve its prognostic value.

## Additional file

**Additional file 1: **Tables S1–S4 and Figures S1–S5.

## Abbreviations

AUC: area under the curve
ICU: intensive care unit
MAP: mean arterial pressure
NIRS: near-infrared spectroscopy
NO: nitric oxide
PU: perfusion units
ROC: receiver operating characteristic
SBF: skin blood flow
SLD: skin laser Doppler
SOFA: sequential organ failure assessment
StO_2_: tissue oxygen saturation
VOT: vascular occlusion test

## References

[1] Sakr Y, Reinhart K, Vincent JL, Sprung CL, Moreno R, Ranieri VM (2006). Does dopamine administration in shock influence outcome? results of the sepsis occurrence in acutely Ill patients (SOAP) study. Crit Care Med.

[2] De Backer D, Biston P, Devriendt J, Madl C, Chochrad D, Aldecoa C (2010). Comparison of dopamine and norepinephrine in the treatment of shock. N Engl J Med.

[3] Weil MH, Shubin H (1971). Proposed reclassification of shock states with special reference to distributive defects. Adv Exp Med Biol.

[4] Vincent JL, De Backer D (2013). Circulatory shock. N Engl J Med.

[5] Edul VS, Enrico C, Laviolle B, Vazquez AR, Ince C, Dubin A (2012). Quantitative assessment of the microcirculation in healthy volunteers and in patients with septic shock. Crit Care Med.

[6] den Uil CA, Lagrand WK, van der Ent M, Jewbali LS, Cheng JM, Spronk PE (2010). Impaired microcirculation predicts poor outcome of patients with acute myocardial infarction complicated by cardiogenic shock. Eur Heart J.

[7] De Backer D, Donadello K, Cortes DO (2012). Monitoring the microcirculation. J Clin Monit Comput.

[8] Lima A, Bakker J (2005). Noninvasive monitoring of peripheral perfusion. Intensive Care Med.

[9] De Backer D, Creteur J, Preiser JC, Dubois MJ, Vincent JL (2002). Microvascular blood flow is altered in patients with sepsis. Am J Respir Crit Care Med.

[10] Trzeciak S, Dellinger RP, Parrillo JE, Guglielmi M, Bajaj J, Abate NL (2007). Early microcirculatory perfusion derangements in patients with severe sepsis and septic shock: relationship to hemodynamics, oxygen transport, and survival. Ann Emerg Med.

[11] De Backer D, Donadello K, Sakr Y, Ospina-Tascon G, Salgado D, Scolletta S (2013). Microcirculatory alterations in patients with severe sepsis: impact of time of assessment and relationship with outcome. Crit Care Med.

[12] De Backer D, Orbegozo CD, Donadello K, Vincent JL (2014). Pathophysiology of microcirculatory dysfunction and the pathogenesis of septic shock. Virulence.

[13] Wright CI, Kroner CI, Draijer R (2006). Non-invasive methods and stimuli for evaluating the skin’s microcirculation. J Pharmacol Toxicol Methods.

[14] Roustit M, Blaise S, Millet C, Cracowski JL (2010). Reproducibility and methodological issues of skin post-occlusive and thermal hyperemia assessed by single-point laser Doppler flowmetry. Microvasc Res.

[15] Doerschug KC, Delsing AS, Schmidt GA, Haynes WG (2007). Impairments in microvascular reactivity are related to organ failure in human sepsis. Am J Physiol Heart Circ Physiol.

[16] Creteur J, Carollo T, Soldati G, Buchele G, De Backer D, Vincent JL (2007). The prognostic value of muscle StO_2_ in septic patients. Intensive Care Med.

[17] Payen D, Luengo C, Heyer L, Resche-Rigon M, Kerever S, Damoisel C (2009). Is thenar tissue hemoglobin oxygen saturation in septic shock related to macrohemodynamic variables and outcome?. Crit Care.

[18] Gomez H, Torres A, Polanco P, Kim HK, Zenker S, Puyana JC (2008). Use of non-invasive NIRS during a vascular occlusion test to assess dynamic tissue O(2) saturation response. Intensive Care Med.

[19] Orbegozo Cortes D, Puflea F, De Backer D, Creteur J, Vincent JL (2015). Near infrared spectroscopy (NIRS) to assess the effects of local ischemic preconditioning in the muscle of healthy volunteers and critically ill patients. Microvasc Res.

[20] Vongsavan N, Matthews B (1993). Some aspects of the use of laser Doppler flow meters for recording tissue blood flow. Exp Physiol.

[21] Levy MM, Fink MP, Marshall JC, Abraham E, Angus D, Cook D (2003). 2001 SCCM/ESICM/ACCP/ATS/SIS international sepsis definitions conference. Crit Care Med.

[22] Knaus WA, Draper EA, Wagner DP, Zimmerman JE (1985). APACHE II: a severity of disease classification system. Crit Care Med.

[23] Vincent JL, Moreno R, Takala J, Willatts S, De Mendonca A, Bruining H (1996). The SOFA (sepsis-related organ failure assessment) score to describe organ dysfunction/failure. On behalf of the working group on sepsis-related problems of the European society of intensive care medicine. Intensive Care Med.

[24] Shapiro NI, Arnold R, Sherwin R, O’Connor J, Najarro G, Singh S (2011). The association of near-infrared spectroscopy-derived tissue oxygenation measurements with sepsis syndromes, organ dysfunction and mortality in emergency department patients with sepsis. Crit Care.

[25] Duret J, Pottecher J, Bouzat P, Brun J, Harrois A, Payen JF (2015). Skeletal muscle oxygenation in severe trauma patients during haemorrhagic shock resuscitation. Crit Care.

[26] Petroni T, Harrois A, Amour J, Lebreton G, Brechot N, Tanaka S (2014). Intra-aortic balloon pump effects on macrocirculation and microcirculation in cardiogenic shock patients supported by venoarterial extracorporeal membrane oxygenation. Crit Care Med.

[27] Taylor WF, Johnson JM, O’Leary D, Park MK (1984). Effect of high local temperature on reflex cutaneous vasodilation. J Appl Physiol Respir Environ Exerc Physiol.

[28] Minson CT, Berry LT, Joyner MJ (1985). Nitric oxide and neurally mediated regulation of skin blood flow during local heating. J Appl Physiol.

[29] Kellogg DL (1985). In vivo mechanisms of cutaneous vasodilation and vasoconstriction in humans during thermoregulatory challenges. J Appl Physiol.

[30] Kellogg DL, Liu Y, Kosiba IF, O’Donnell D (1985). Role of nitric oxide in the vascular effects of local warming of the skin in humans. J Appl Physiol.

[31] Kellogg DL, Zhao JL, Wu Y (1985). Roles of nitric oxide synthase isoforms in cutaneous vasodilation induced by local warming of the skin and whole body heat stress in humans. J Appl Physiol.

[32] Vaughn MW, Kuo L, Liao JC (1998). Effective diffusion distance of nitric oxide in the microcirculation. Am J Physiol.

[33] Kim-Shapiro DB, Schechter AN, Gladwin MT (2006). Unraveling the reactions of nitric oxide, nitrite, and hemoglobin in physiology and therapeutics. Arterioscler Thromb Vasc Biol.

[34] Lupp C, Baasner S, Ince C, Nocken F, Stover JF, Westphal M (2013). Differentiated control of deranged nitric oxide metabolism: a therapeutic option in sepsis?. Crit Care.

[35] Cauwels A (2007). Nitric oxide in shock. Kidney Int.

[36] Hollenberg SM, Cinel I (2009). Bench-to-bedside review: nitric oxide in critical illness—update 2008. Crit Care.

[37] Alexander JH, Reynolds HR, Stebbins AL, Dzavik V, Harrington RA, Van de Werf F (2007). Effect of tilarginine acetate in patients with acute myocardial infarction and cardiogenic shock: the TRIUMPH randomized controlled trial. JAMA.

[38] Engelberger RP, Pittet YK, Henry H, Delodder F, Hayoz D, Chiolero RL (2011). Acute endotoxemia inhibits microvascular nitric oxide-dependent vasodilation in humans. Shock.

[39] Ninet J, Fronek A (1985). Cutaneous postocclusive reactive hyperemia monitored by laser Doppler flux metering and skin temperature. Microvasc Res.

[40] Metzler-Wilson K, Kellie LA, Tomc C, Simpson C, Sammons D, Wilson TE (2012). Differential vasodilatory responses to local heating in facial, glabrous and hairy skin. Clin Physiol Funct Imaging.

[41] Del Pozzi AT, Hodges GJ (2015). To reheat, or to not reheat: that is the question: the efficacy of a local reheating protocol on mechanisms of cutaneous vasodilatation. Microvasc Res.

[42] Roustit M, Millet C, Blaise S, Dufournet B, Cracowski JL (2010). Excellent reproducibility of laser speckle contrast imaging to assess skin microvascular reactivity. Microvasc Res.

[43] Bezemer R, Klijn E, Khalilzada M, Lima A, Heger M, van Bommel J (2010). Validation of near-infrared laser speckle imaging for assessing microvascular (re)perfusion. Microvasc Res.

[44] Pauling JD, Shipley JA, Raper S, Watson ML, Ward SG, Harris ND (2012). Comparison of infrared thermography and laser speckle contrast imaging for the dynamic assessment of digital microvascular function. Microvasc Res.

