# Supplementary material for: Skin microcirculatory reactivity assessed using a thermal challenge is decreased in patients with circulatory shock and associated with outcome
Source: Ann Intensive Care. 2018 May 3;8:60. doi: 10.1186/s13613-018-0393-7 (PMC5934288; doi:10.1186/s13613-018-0393-7)
Supplement: Supplementary file 1 — Additional file 1: Tables S1–S4 and Figures S1–S5. [file 13613_2018_393_MOESM1_ESM.pdf]

Skin Microcirculatory Reactivity Assessed using a Thermal Challenge is Decreased in Patients with Circulatory Shock and Associated with Outcome

Orbegozo et al

Additional File 1

Figure S1. Schematic representation of a thermal challenge during skin blood flow (SBF) monitoring with skin laser Doppler (SLD). Positions for the probes in healthy volunteers are marked as A (forearm) and B (palm). In patients with circulatory shock, all measurements were performed at A.

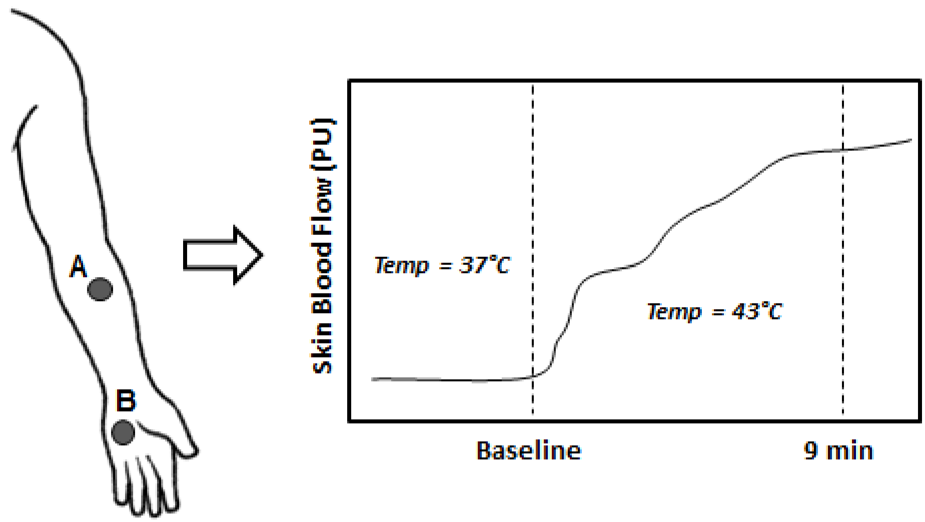

Table S1. Main characteristics of healthy volunteers

Variable	Result
Subjects [n]	18
Male [n (%)]	12 (67)
Age (years)	39 ± 12
Body mass index (Kg/m^2)	24 ± 3
Heart rate (beats per min)	68 ± 7
Mean arterial pressure (mmHg)	88 ± 10
SpO_2 (%)	98 ± 2

Table S2. Comparison of skin laser Doppler (SLD) variables in the healthy volunteers on the forearm and hand

SLD Variable	Forearm	Hand	P
Skin Blood Flow 37°C (PU)	10.3 (8.0-14.2)	94.5 (57.2-130.0)	<0.01
Skin Blood Flow 43°C (PU)	112.8 (88.0-150.0)*	201.5 (132.0-255.0)*	<0.01
Skin Blood Flow Ratio	11.2 (9.4-13.4)	2.0 (1.7-2.6)	<0.01

* = $p < 0.05$ compared with baseline values at 37°C

Figure S2. Comparison of the skin blood flow (SBF) ratio after 48 hours in 20 patients

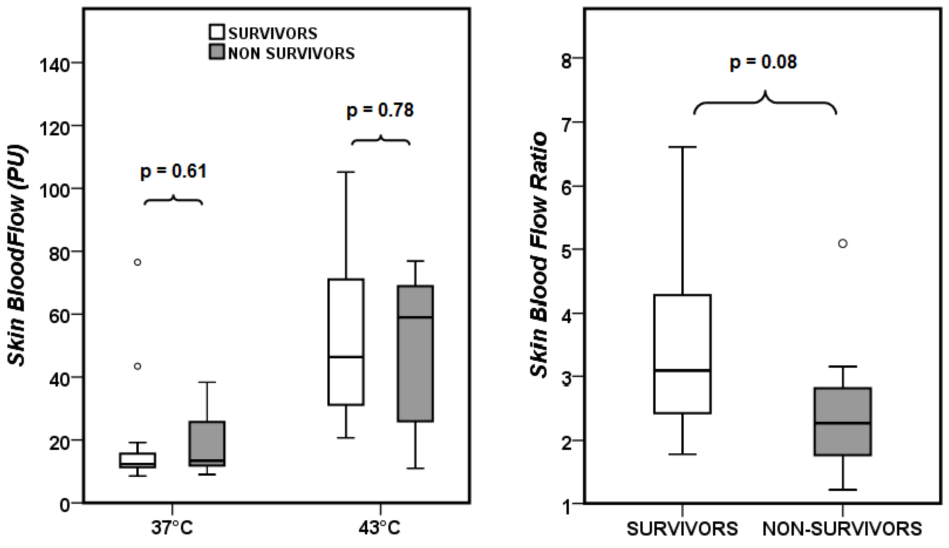

Figure S3. Comparison of the evolution between T0 and T48 in the 20 patients with two measurements (p values calculated using a 2 way ANOVA analysis)

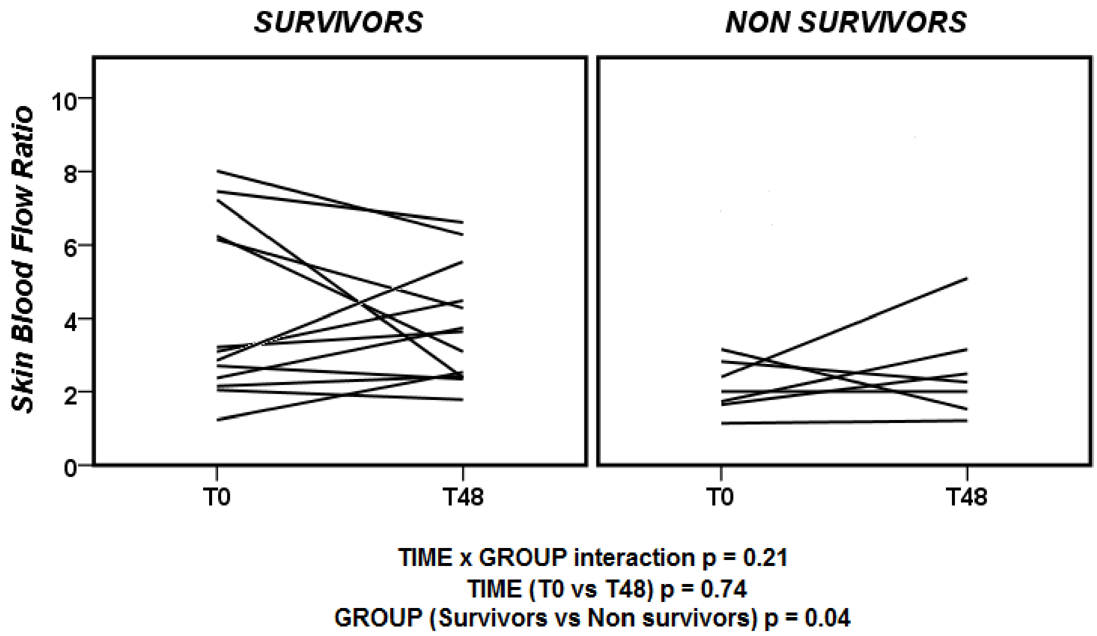

Figure S4. Correlation of norepinephrine dose and arterial lactate concentration with the skin blood flow (SBF) ratio in the patients with circulatory shock.

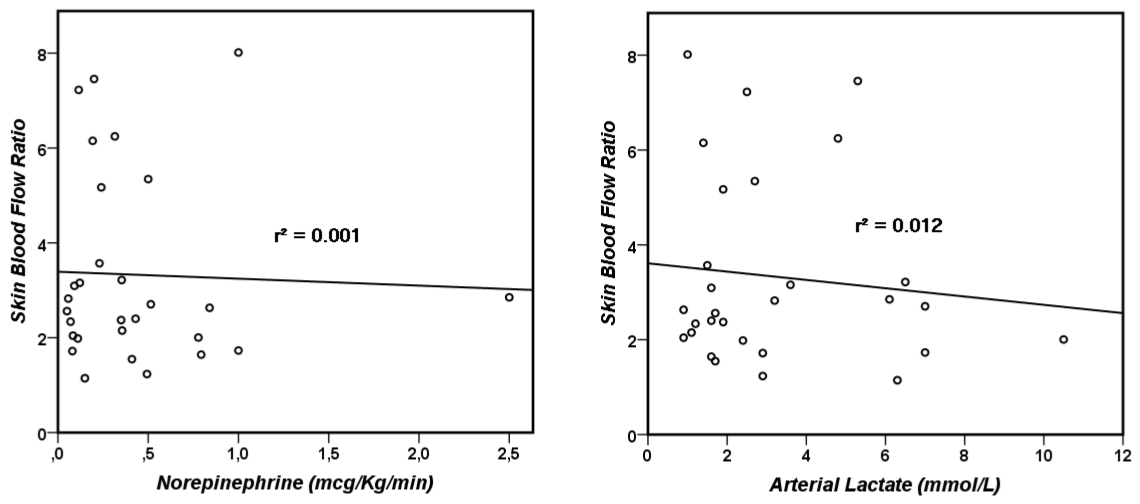

Table S3. Comparison of skin laser Doppler (SLD) variables in septic and non-septic patients

	INITIAL COHORT			CONFIRMATORY COHORT		
SLD Variable	SEPSIS (n=15)	NO SEPSIS (n=14)	P	SEPSIS (n=28)	NO SEPSIS (n=7)	P
Skin Blood Flow 37°C (PU)	13.6 (12.2-22.8)	11.7 (10.0-19.5)	0.69	12.9 (8.6-18.3)	13.0 (9.7-22.5)	0.81
Skin Blood Flow 43°C (PU)	39.0 (25.7-76.9)	34.4 (23.8-77.1)	0.91	40.4 (22.2-54.5)	41.5 (31.7-80.3)	0.34
Skin Blood Flow Ratio	2.4 (2.0-5.3)	2.8 (1.7-3.6)	0.83	2.6 (1.9-4.3)	3.3 (1.8-3.7)	0.43

Figure S5. Correlation of central, forearm (before heating to 37°C) and central minus forearm temperature with the skin blood flow (SBF) ratio in the patients with circulatory shock

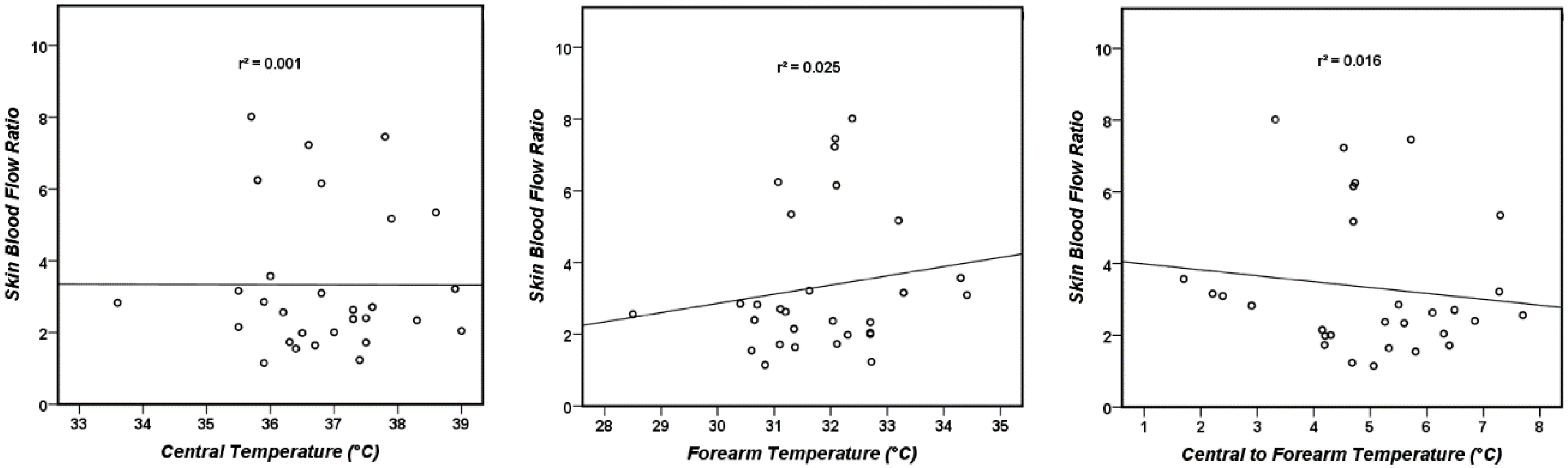

Table S4. Univariate and multivariable analysis of variables to predict ICU mortality

Type of analysis	Variable	OR	95% CI	P
Univariate analysis	Lactate	1.259	0.903-1.753	0.174
	Mean arterial pressure	1.024	0.964-1.089	0.438
	Norepinephrine dose	5.451	0.530-56.077	0.154
	Sepsis	1.200	0.267-5.400	0.812
	SOFA score	1.344	1.001-1.805	0.049
	Skin blood flow ratio	0.447	0.192-1.039	0.061
	APACHE II score	1.149	0.999-1.320	0.051
Multivariable analysis (Model 1)	SOFA score	1.161	0.772-1.747	0.473
	Skin blood flow ratio	0.122	0.015-0.993	0.049
	APACHE II score	1.369	1.033-1.815	0.029
Multivariable analysis (Model 2)	Skin blood flow ratio	0.123	0.015-0.981	0.048
	APACHE II score	1.386	1.059-1.813	0.017

Model 1: Considers all variables with p < 0.1 in the univariate analysis. [Consider all variables simultaneously].
Model 2: Considers all variables with p < 0.1 in the univariate analysis and also other relevant clinical and hemodynamic variables (lactate, mean arterial pressure, norepinephrine dose, sepsis). [Backward stepwise selection and removal of variables based on the probability of the likelihood ratio statistic].
